# An overview of reliable and representative DVC measurements for musculoskeletal tissues

**DOI:** 10.1111/jmi.70008

**Published:** 2025-07-10

**Authors:** Gianluca Tozzi, Enrico Dall'Ara

**Affiliations:** ^1^ Centre for Advanced Manufacturing and Materials School of Engineering, University of Greenwich London UK; ^2^ Division of Clinical Medicine, Mellanby Centre for Musculoskeletal Research University of Sheffield Sheffield UK; ^3^ Insigneo Institute University of Sheffield Sheffield UK

**Keywords:** biomaterials, digital volume correlation, in situ mechanics, musculoskeletal tissues

## Abstract

Musculoskeletal tissues present complex hierarchical structures and mechanical heterogeneity across multiple length scales, making them difficult to characterise accurately. Digital volume correlation (DVC) is a non‐destructive imaging technique that enables quantification of internal 3D strain fields under realistic loading conditions, offering a unique tool to investigate the biomechanics of biological tissues and biomaterials. This review highlights recent advancements in DVC, focusing on its applications across scales ranging from organ‐ to tissue‐level mechanics in both mineralised and soft tissues. Instead of a traditional systematic review, we identify key technical challenges including the treatment of tissue interfaces, border effects, and the quantification of uncertainty in DVC outputs. Strategies for improving measurement accuracy and reliability are discussed. We also report on the increasing use of DVC in in vivo applications, its coupling with computational modelling to inform and validate biomechanical simulations, and its recent integration with data‐driven methods such as deep learning to directly predict displacement and strain fields. Additionally, we examine its application in tissue engineering and implant–tissue interface assessment. By addressing such areas, we outline current limitations and emerging opportunities for future research. These include advancing precision, enabling clinical translation, and leveraging machine learning to create more robust, automated, and predictive DVC workflows for musculoskeletal health and tissue engineering.

## 1 INTRODUCTION

The Digital Volume Correlation (DVC) technique is the only experimental method capable of characterising local internal deformation fields of both natural and engineered biomaterials under complex loading conditions. For a comprehensive overview of the literature, readers are encouraged to explore other review articles on in situ mechanical testing combined with Synchrotron^1^ and desktop^2^ imaging, and those more specific for DVC applications, such as those by Roberts et al.^3^ and Grassi and Isaksson^4^ on bone deformation, and by Dall'Ara and Tozzi^5^ on different musculoskeletal tissues. This review aims to focus on recent advancements and applications of DVC, offering guidelines to emphasise its potential while addressing the challenges and risks associated with the use of this technology.

The DVC approach was initially developed in the late 1990s for assessing the deformation in trabecular bone tissue, a complex material for which full‐field internal strains under load could not be assessed with any other technique.^6^ Two microcomputed tomography (microCT) images of the undeformed and deformed trabecular bone specimens before and after a stepwise compression were correlated, leading to the first experimental measurement of local bone displacements and strains. Already at that time, the potential of the DVC in assessing internal strains and in validating the prediction of local deformation by computational models became apparent.^7^ Nevertheless, since then and after a development break of almost a decade, the approach has grown and has been used to characterise the mechanical properties of different natural and engineered biomaterials, at different dimensional scales, with a particular emphasis on musculoskeletal tissues.^5^ Multiple DVC approaches have been developed (i.e., local, global, combined approaches) to measure more accurately the local deformation in different types of structures, and in some cases, probably too few, the results obtained using different approaches on the same dataset have been compared to highlight their complementarity.^8^, ^9^, ^10^, ^11^ Moreover, since the early 2000s, DVC outputs have been extensively used to validate the results of computational models that aim at predicting the biomechanics of biological structures of increased complexity.^12^, ^13^, ^14^, ^15^ Although the growing adoption of this approach by the research community over the past decade highlights its potential, it is crucial to consider how DVC should be applied. It's important to determine what should be reported in each study to ensure the reliability of the results for specific applications, as well as to establish guidelines for reporting findings in a way that avoids speculation and over‐interpretation.

The aim of this review paper is to highlight a selection of DVC applications found in the literature, focusing on key examples and reflecting on the most recent developments, rather than providing a systematic review of the topic. We believe these discussions and guidelines are essential for the continued growth of this research area, which seeks to enhance experimental assessments of internal displacements and strains in biological tissues and biomaterials under increasingly complex loading scenarios.

## 2 DVC ANALYSES ACROSS THE DIMENSIONAL SCALES FOR MINERALISED AND SOFT TISSUES

**FIGURE 1:**
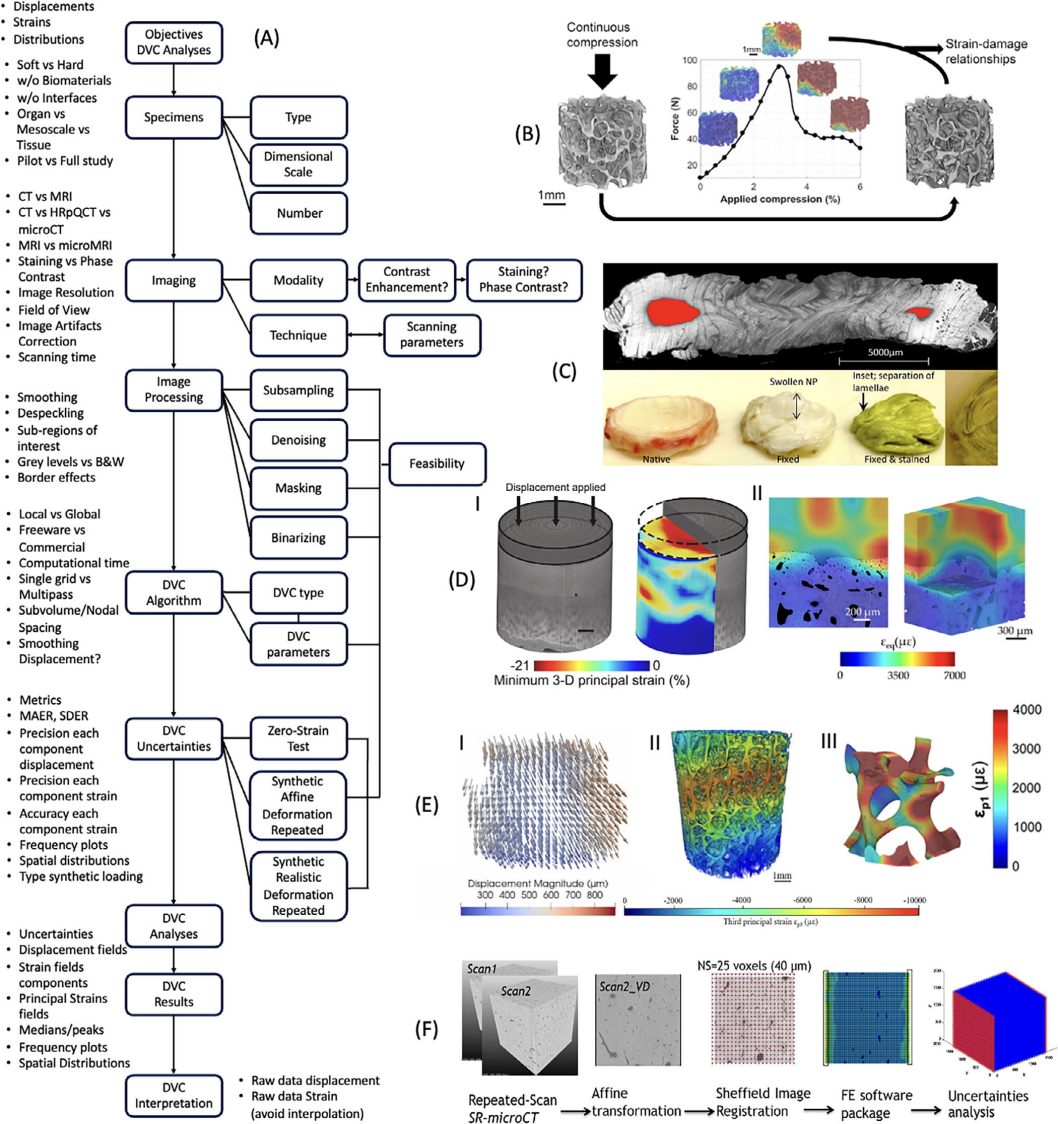
(A) Schematic workflow addressing some of the main features of the pipeline to perform DVC analyses in musculoskeletal tissues. (B) Experimental overview for the deformation of bone tissue using in situ SR‐microCT compression coupled with DVC.^16^ (C) Fixing and staining increases X‐ray contrast but fails to fully penetrate the whole intervertebral disc and causes major structural changes.^17^ (D) (I) 3D strain distribution of human articular cartilage following tissue staining^18^ and at (II) the cartilage–bone interface of bovine osteochondral plugs in unstained tissue and with phase‐contrast microCT.^19^ (E) (I) Displacement field magnitude and distribution for in situ XCT of an intact vertebra using local DVC approach.^20^ (II) Strain field interpolation (i.e., subvolumes including trabeculae) from Ref. (21) and (III) measurement at tissue level (i.e., subvolumes included in trabeculae) from Ref. (22). (F) General sequence used to investigate the precision and accuracy of global DVC approach for measurement of strains in cortical bone and including synthetically deformed images, a deformable registration, the registration grid with conversion to a finite element (FE) model to compute the strain field as well as a custom‐made script to plot uncertainties.^23^

DVC has been applied to assess the full field three‐dimensional internal deformation of biological tissues at different dimensional scales. Depending on the image modality used, DVC analyses can characterise the properties of hard mineralised tissues: bones and teeth scanned with Computed Tomography (CT), microCT or Synchrotron microCT (SR‐microCT); soft tissues such as intervertebral discs, meniscus or cartilage scanned with Magnetic Resonance (MR) imaging, contrast enhanced microCT or phase‐contrast microCT (Figure 1A).

### 2.1 Organ level

Starting from the largest dimensional scale, the deformation of human organs or big portions of them have been analysed with DVC. For example, deformation of the whole human proximal femur has been measured by using high‐resolution (SR‐microCT, 120 µm)^24^ or low‐resolution (clinical CT, ∼1000 µm)^25^ input images. These studies have characterised the failure behaviour of the femur replicating different loading conditions including one‐legged stance or fall on the side. For the first time the failure mechanisms of the bone have been assessed in detail, highlighting how tensile and shear strains in the sub‐capital cortical and trabecular bone of the proximal femur lead to crack initiation. Martelli et al.^24^ have also highlighted the challenges in performing DVC analyses starting from high‐resolution images (impressively 30 µm voxel size for the whole proximal femur scanned with SR‐microCT^26^) of such a big portion of the bone. They proposed a two‐scale approach to identify first the region of localisation of the deformation from a subsampled image (120 µm) of the whole scanned proximal femur, and then assessing the detailed deformation running the DVC analyses on full resolution (30 µm) images cropped around the region of interest.^24^ On the other hand, considering the relatively small size of vertebrae and spine segments, DVC has been successful in characterising the effect of microstructure,^27^ loading conditions,^28^ metastatic lesions,^29^ and intervertebral disc degeneration^30^ on the local deformation and/or failure behaviour of the vertebral body. These studies have showcased the potential of DVC in identifying how the very complex and heterogeneous internal microstructure of the human vertebral body, which includes a core of trabecular bone enclosed within thin cortical endplates and shell, fails under load. They have also highlighted the importance of studying what happens at the interface between the intervertebral disc and the vertebral body's endplates, something neglected in previous experimental spine biomechanics studies.^31^ In other cases, DVC has been used to study the deformation of complex shapes and structures where the application of strain gauges or Digital Image Correlation (DIC) would have been very challenging,^32^ for example big portions of the human scapula.^14^ The complication of designing the loading conditions for such a complex structure was overcome in that study by using a six degrees of freedom hexapod within an industrial microCT system and acquiring the images of the bone only around the loaded structure.

While all previous studies have employed CT (from low‐resolution clinical CT to very high‐resolution SR‐microCT) the DVC approach has been recently applied to Magnetic Resonance Imaging (MRI) to evaluate the deformation in soft organs. For example, the effect of the degeneration of the IVD has been studied using a combination of DVC and MRI imaging.^33^, ^34^ In the first study the authors had to overcome challenges in the design of 9.4T MRI‐compatible loading device, but the obtained ex vivo measurements on cadaver tissues have enabled the assessment of the IVD deformation in healthy, degenerated and treated IVDs, highlighting the potential of different biomaterials to restore the IVD functionality. Another application from the same group challenged the DVC approach to perform measurements in vivo, by leveraging on the absence of ionising radiation from 3T MRI imaging.^35^ In that case the high deformation measured in the soft IVD reduced the effect of the high uncertainties in the DVC approach due to the relatively low resolution in MRI imaging. In another study MRI‐driven DVC has been used to evaluate deformation of the human buttock during sitting.^36^ In this case the DVC results were used to quantify the slide of the gluteus maximus away from the ischial tuberosity during sitting, providing an approach that could be employed in ergonomic design.

Studying large portions of organs with DVC presents several challenges: (1) Achieving the optimal compromise between field of view, image resolution, and DVC calculation time is difficult. While multirun approaches have proven effective in addressing this challenge, they can only be applied when the deformation is localised to a specific subregion of the specimen. (2) When organs are composed of tissues with significantly different densities and radio transparencies (e.g., vertebral bodies and intervertebral discs in spine segments), it becomes challenging to visualise the necessary features for accurate DVC outcomes using a single technique. Contrast enhancement methods are commonly used to improve feature visibility but may alter the properties of stained tissues. Phase contrast imaging has been used to overcome this issue, but it is usually still limited to small portions of the specimens. DVC approaches based on multimodal imaging hold great potential but remain largely unexplored. (3) Complex organs often have larger, more convoluted external surfaces that lack distinctive features, making it difficult for local and global DVC methods to converge properly, which reduces accuracy in those areas.

### 2.2 Mesoscale and tissue level

At the mesoscale (between the organ and the tissue dimensional scales), the DVC approach has been employed to understand the functionality of the organ considering the underneath tissue microstructure. For example, the effect of degeneration of the local microstructure due to osteoarthritis in the human femoral head has been evaluate in a pilot study from Ryan et al.,^37^ who linked the heterogeneity of the tissue to atypical localisation of the strains around features induced by the disease. As with most DVC studies at the mesoscale (Figure 1B), the generalisation of results is limited by the small number of specimens tested. One major challenge of using DVC at this scale is the high cost and time required for testing and imaging specimens, which makes it impractical for large sample sizes. Additionally, while high‐resolution imaging can enable the identification of numerous features distributed heterogeneously within the specimens (e.g., cortical bone, trabecular bone, disease‐related features, or treatment effects), the large tissue volumes involved result in long computation times, even when high‐performance computing is used. Furthermore, most commercially available and open‐source DVC software does not support parallel processing on servers, limiting the ability to efficiently process large, high‐resolution images.^24^

The application of DVC, firstly introduced to evaluate the effect of heterogeneous deformations in trabecular bone tissue, has been followed by a strong focus on increasing the input image resolution in order to improve the accuracy of the DVC approach for the assessment of the strains in local bony features like trabeculae and osteons.^38^, ^39^, ^40^ Nevertheless, in the past 10 years the DVC approach has been adapted to analyse complex soft tissues by processing: (1) contrast‐enhanced microCT images; (2) phase‐contrast microCT images; and (3) high‐resolution MRI images. In a recent study, the deformation of the muscle‐tendon junction of wild‐type mice under tensile loading has been studied by a combination of DVC and contrast enhanced microCT imaging, providing more insights on the mechanisms of injury in this complex structure.^41^ However, as staining clearly showed important morphological alterations in soft tissues^17^ (Figure 1C), it is unclear how the protocol used (1% phosphotungstic acid in 70% ethanol solution) also affects their mechanics, making it difficult to interpret the obtained results. To overcome the limitation of using contrast agents that may affect the mechanical properties of the tissue of interest^18^, ^42^, ^43^ (Figure 1D, I), phase contrast microCT imaging has been used in combination with DVC to study the deformation in soft tissues (Figure 1D, I). Tozzi et al.^19^ explored the use of propagation‐based phase‐contrast microCT imaging on the cartilage–bone interface of osteochondral specimens from bovine joints (Figure 1D, II). This approach was associated with reasonable uncertainties thanks to the visualisation of chondrocytes lacunae in the cartilage and enabled the determination of residual strains induced by the mechanical testing and the compliance of the bone–cartilage interface. Similarly, Disney et al.^44^ used phase contrast SR‐microCT to characterise the deformation in rat IVD by mechanically testing the whole spine segments. The small size of the specimen and the high flux of the synchrotron light enabled the visualisation of the IVD fibres, which allowed the authors to identify the localisation of strain patterns in the extracellular matrix of the IVD. These studies have been pivotal in confirming the potential of phase‐contrast microCT‐ and SR‐microCT‐based DVC analyses, and highlighted the current limitations of the approach, which requires further developments to better understand the deformation of soft tissues. On the other hand, loading devices have been redesigned to make them compatible for micro‐MRI, enabling the characterisation of soft tissues. For example, this approach has been found suitable to characterise the displacement of the human meniscus during the compression of the knee in a pilot study on two cadaver healthy and injured knees,^45^ highlighting the potential of future applications in weight bearing conditions in patients. These applications at the lower dimensional scale highlight the need of reducing the uncertainties of the DVC approach to assess strain in smaller parts of the considered tissues. Considering the limited resolution achievable with the current MRI technologies, the approach so far has been to image the tissues with SR‐microCT or adding contrast agents. While this approach has the potential of better understanding the deformation of the main components of biological tissues, it is paramount to avoid damaging the tissue with ionising radiation or with aggressive chemicals. Further development in all three areas mentioned above are required to improve current assessment of the deformation in soft tissues.

## 3 DVC MEASUREMENTS OF REALISTIC DEFORMATIONS

### 3.1 Visualisation and interpretation of the measurements

DVC calculates displacements from the correlation of subvolumes (or 3D subsets) between the reference and deformed volumetric images (Figure 1E, I). Therefore, the spatial resolution of the measurement strictly relies on the number of subvolumes used for the correlation which individually must contain enough quality of greyscale information, irrespective of the typology of DVC strategy. In local DVC (and more in general DIC^32^) one of the strategies to ‘increase’ the number of measurement points is to make use of overlapping subvolumes. However, the use of subvolume overlap in DVC was found to produce an important increase in strain uncertainties which may jeopardise application in biological tissues.^46^

In the realm of applications for the characterisation of musculoskeletal tissues, appropriate subvolume size is selected to target a specific dimensional scale, that is, organ or tissue level, ensuring a reasonable trade‐off between spatial resolution of the measurement and displacement/strain uncertainties. In the case of bone, DVC was used to compute strain fields in structural elements (e.g., vertebral bodies^47^) as well as their trabecular and cortical microstructures.^16^ One important aspect to consider here is the amount and nature of features available within the subvolume to justify a specific length scale. For example, if the reference features is a number of trabeculae, we can interpret that measurement as representative of apparent level mechanics^21^ (Figure 1E, II). In order to claim measurements at the tissue level, which can be the case of a single trabecula, we must ensure that smaller and resolvable features (i.e., osteocyte lacunae) are visible and that a sufficient number of subvolumes (up to 3–4) is included across the trabecular thickness^22^ (Figure 1E, III). The identification of more features in the image is obviously related to system capability and image signal‐to‐noise ratio to achieve the desired spatial image resolution, which will dictate the amount of subvolumes and independent measurement points for DVC. In fact, even in the case of small voxel size the ability to discriminate features of interest for DVC could be compromised and quality enhancement via image post‐processing (i.e., contrast, sharpness, filtering) may be required. In such a situation, the resulting image correlation could introduce artefacts in the measurement.^40^ Thus, it is recommended that the scale of investigation targeted when using DVC would be driven by the actual nature of resolved features in the image (or greyscale gradients in the region of interest) and density of measurement achievable in the biological structure, rather than linearly or nonlinearly interpolating between fewer data points, which can only give an indication of displacement/strain levels in regions with no actual measurement when overlaid to the greyscale image texture.^48^ Although this procedure is useful to identify strain gradients within the tissue structure, it is good practice to indicate this ‘interpolated’ map as strain visualisation. Thus, avoiding subsequent claims of tissue‐level strain determination which barely include a single sub‐volume measurement.

### 3.2 Treating interfaces and border effects

Accurate DVC analysis of muscoloskeletal tissues requires careful attention to interface and border effects, which can introduce significant errors if not properly addressed. These challenges arise due to the heterogeneous, anisotropic nature of these tissues and the sharp transitions between different tissue types, such as bone–cartilage or bone–tendon interfaces. Moreover, the sharp gradients in grey levels at the border of the tissue/organ to be analysed are usually increased when they are dissected from the body in ex vivo experiments. Considering these effects is crucial for obtaining reliable measurements of mechanical behaviour and understanding tissue function under physiological loads.

Border effects in DVC measurements are primarily caused by the lack of sufficient correlation information near the edges of the region of interest (ROI).^9^, ^49^ This limitation is due to the fact that the correlation algorithm relies on voxel subvolumes within the 3D image, and subvolumes located near tissue boundaries may lack complete data, leading to reduced accuracy and increased noise in the strain fields. In musculoskeletal tissues, border effects can be particularly problematic because these tissues often have complex geometries with irregular, non‐uniform borders.^11^ Strategies to mitigate border effects include padding the image volume with synthetic data, using adaptive subvolume selection techniques, and carefully defining the ROI to avoid areas with insufficient data. Another common approach is to apply smoothing filters or regularisation methods to reduce noise at the borders,^40^ though these techniques must be used cautiously to avoid over smoothing and loss of detail in strain maps.

Interface effects occur at the boundaries between different tissue types with distinct mechanical properties and imaging contrast. For example, at the bone‐implant interface, the sharp transition in stiffness and density can lead to artificial strain concentrations and erroneous displacement measurements. Moreover, different tissues and biomaterials usually have very different patterns of features, which drive the accuracy and precision of the DVC (see Section 4), making it difficult to know the DVC uncertainties at the interfaces. These effects can obscure the true mechanical response and make it difficult to distinguish between genuine tissue deformation and artefacts.^50^ Proper treatment of interface effects requires advanced image preprocessing and segmentation techniques to accurately delineate tissue boundaries before DVC analysis. Additionally, advanced synergies between finite element (FE) analysis and DVC incorporating tissue‐specific mechanical properties into the correlation process, can reduce the likelihood of artificial strain concentrations at interfaces.^51^

High‐quality imaging data is essential for minimising both border and interface effects. MicroCT and SR‐microCT imaging provide high‐resolution datasets suitable for DVC analysis of musculoskeletal tissues.^1^ The choice of imaging modality should balance resolution, field of view, and contrast between tissue types to ensure accurate correlation, ensuring a reduced tissue irradiation and degeneration. Recent advances in computational methods offer promising solutions to border and interface challenges. Deep learning‐based segmentation algorithms have shown potential for improving tissue boundary detection and reducing artefacts at interfaces.^52^ Such machine learning models can subsequently correct border errors, improving the reliability of DVC measurements. In conclusion, addressing border and interface effects is critical for ensuring accurate and reliable DVC measurements in musculoskeletal tissues. Effective solutions include advanced image preprocessing and the integration of tissue‐specific models. Continued research in machine learning and multimodal imaging holds great promise for enhancing the accuracy and applicability of DVC in complex biological systems.

### 3.3 Potential and limitations of in vivo DVC measurements

Weight‐bearing CT (WBCT) enables the assessment of joint mechanics under physiological load conditions, offering a more accurate representation of in vivo biomechanics compared to non‐weight‐bearing imaging. DVC applied to WBCT data has been instrumental in quantifying internal deformations within bone structures during load‐bearing activities. For instance, Peña Fernández et al.^53^ successfully combined image‐based DVC analysis with WBCT to calculate the kinematics of the subtalar joint during inversion and eversion motions, achieving a precision for displacement measurements ranging from 20 to 250 µm and providing a comprehensive understanding of joint function and potential pathological changes.

MRI offers superior soft tissue contrast without ionising radiation, making it suitable for assessing soft tissues like intervertebral discs (IVDs). Combining DVC with high‐field MRI has enabled the measurement of internal strains within IVDs, providing insights into their mechanical behaviour under various loading conditions. Tavana et al. developed a methodology using DVC based on MRI to assess internal deformations in human IVDs, highlighting the potential of this technique for non‐invasive evaluation of spinal biomechanics.^35^ This approach enables the assessment of internal strain distributions within the IVDs, which is crucial for understanding the progression of degenerative IVD diseases and evaluating the efficacy of therapeutic interventions. Further advancements in this field include the work by Rahman et al., who utilised DVC combined with ultra‐high‐resolution MRI to quantify internal IVD strains and assess nucleus replacement device designs.^33^ Their study demonstrated the feasibility of using DVC with high‐field MRI to evaluate internal strains within the IVDs, providing valuable insights into the mechanical behaviour of the disc and the potential impact of nucleus replacement devices. Additionally, Tavana et al. explored the application of DVC in combination with 3T clinical MRI to assess deformations and strains in human bones in vivo.^54^ This study demonstrated the feasibility of using DVC with clinical MRI to evaluate internal bone mechanics non‐invasively, providing a potential tool for clinical assessments of bone health and disease progression. In another study, the DVC has been applied to proton density MR images of the human heel soft tissues in vivo, measuring the deformation of the fat pad under load and informing computational models for the prediction of the deformation in soft tissues.^55^

Despite these advancements, several challenges persist in applying DVC to WBCT and MRI data. One significant challenge is the trade‐off between spatial resolution and imaging speed, which can affect the accuracy of strain measurements. Additionally, patient motion during imaging can introduce artefacts, which would affect DVC uncertainties and the interpretation of the results. Future research should focus on developing faster, motion‐robust imaging protocols and refining DVC algorithms to enhance strain quantification in both bone and soft tissues. Moreover, integrating DVC with advanced imaging techniques, such as ultra‐high‐field MRI and dynamic CT, could provide more detailed insights into tissue mechanics under physiological loading conditions.

## 4 LATEST APPLICATIONS AND CURRENT TRENDS

### 4.1 DVC uncertainties and optimisation: from zero‐strain analyses to realistic (synthetic) loading

For the accurate interpretation of DVC results, it is crucial to assess its precision and accuracy. While this is straightforward for most standard measurement methods, it is not as clear‐cut for DVC applications. The challenge lies in the absence of more accurate reference methods for estimating 3D displacement and deformation fields, with which DVC results can be directly compared.

Moreover, understanding and quantifying the uncertainties in DVC measurements is essential, not only to identify these uncertainties but also to determine the optimal measurement pipeline (image acquisition, image processing, selection of DVC parameters) to achieve the most accurate results. The primary challenge is identifying a scenario where both the inputs (images, DVC parameters) and outputs (displacement and strain fields) are known, enabling error calculation for the DVC measurements. The uncertainties in DVC measurements have typically been assessed using a zero‐strain scenario. This approach, first introduced by Liu and Morgan^56^ for various types of trabecular bone tissues, assumes that when analysing two repeated scans, the strain field remains unchanged, either zero everywhere or constant between unloaded and loaded configurations. This method has been widely used in musculoskeletal applications^5^ and recently also to test the feasibility of using high‐resolution peripheral quantitative CT (HR‐pQCT) imaging to evaluate the deformation of the human femur ex vivo.^57^ While this approach offers a useful initial estimate of the trade‐off between precision in strain measurements and spatial resolution,^58^ it is limited by the simplification of assuming homogeneous, zero deformations across the entire specimen. Given that the strength of the DVC method lies in its ability to capture 3D deformation fields in complex, heterogeneous tissues, the uncertainties derived from a zero‐strain scenario may be underestimated compared to more realistic, complex loading conditions. Despite this limitation, this approach has demonstrated that uncertainties can vary for different components of strain^46^ and that the microstructure of the specimen may influence the uncertainty levels.^59^ Therefore, it is essential to test the uncertainties of the DVC approach across various specimens and strain components to better understand its reliability for each specific application.

As an alternative to zero‐strain scenarios, synthetic deformations have been applied to input images to evaluate the algorithm's ability to estimate strain fields resulting from displacements or homogeneous deformations. While this method was widely used in the early development of DVC techniques for bone applications,^60^ it is important to note that testing DVC uncertainties by inputting one image and its synthetically deformed counterpart may be misleading. This is because such images do not account for random image noise between the two input images, a major factor that introduces errors in the DVC measurement pipelines.^61^ Even more problematic is that the image noise in both the undeformed and synthetically deformed images forms a pattern of features that aids in assessing the displacement and strain fields, much like the speckle patterns used in DIC measurements. As a result, this approach tends to underestimate DVC uncertainties and should be avoided.

To advance the assessment of DVC uncertainties in more realistic scenarios, the application of synthetic deformations to repeated images has been introduced^23^, ^37^ (Figure 1F). This method combines the techniques mentioned earlier, where the input data for DVC consists of the undeformed image and the repeated image (from a zero‐strain scenario) to which a synthetic deformation field is applied. In this case, DVC uncertainties can be evaluated for a simplified deformation field (usually homogeneous compression, tension, or shear by transforming the repeated image with an affine transformation) rather than a zero‐strain condition. While this approach has recently been used to evaluate the deformation of bone tissue at various dimensional scales, it has not yet been systematically applied to soft tissues or more complex structures. Even though the deformation field is still simplified compared to the realistic loading scenarios that will be investigated with the DVC, adopting this method is recommended for testing future DVC applications and better understanding the effect of the chosen DVC parameters on the final outcomes.

Finally, in a recent study, a synthetic deformation based on a biomechanical computational model has been proposed to assess the performance of different DVC algorithms in estimating complex, heterogeneous deformation in the human femur.^8^ Briefly, in that study, deformed images were generated by warping undeformed images with a displacement field computed from a finite element simulation based on the undeformed image. By scaling the obtained displacement field, different synthetically deformed images were created using different displacement magnitudes. Both the undeformed and synthetically deformed images were then input into the DVC, and the results were compared with the known displacement and strain fields used to generate the synthetically deformed images. While this approach provides a heterogeneous and realistic deformation field, that pilot study applied the known displacement field on the input image (instead of the repeated image as suggested earlier), which reduced the influence of potential image noise in the final calculations. However, it should be noted that the heterogeneous deformation field applied to the undeformed image, along with the resulting heterogeneous image interpolation, introduces a form of heterogeneous (but not random) noise into the process. Despite this, future applications that use synthetically deformed repeated images with deformations estimated from biomechanical models have the potential to further challenge DVC accuracy and should be explored. However, it is important to note that applications involving soft tissues remain more challenging due to the complexity of biomechanical models and constitutive laws in these cases.

### 4.2 DVC to inform and validate finite element models

Coupling DVC with biomechanical models, such as the micro‐Finite Element (microFE) approach, has the potential to significantly enhance our understanding of complex biological tissues. Developed in the mid‐1990s, the microFE approach converts segmented microCT images into structural models to predict deformation at the voxel level.^62^ In contrast, DVC is inherently limited in assessing local deformations within individual structural components of the tissue being studied (e.g., single trabeculae in trabecular bone or individual fibres in intervertebral discs). As mentioned above, this limitation arises from the lack of sub‐structural image features necessary for driving the image correlation, with DVC accurately estimating local deformation only at the 40–50 voxel scale.^11^ As a result, using DVC image processing alone does not provide accurate assessments of deformation in these sub‐structures. However, biomechanical models can help estimate such deformations by incorporating information about the local mechanical properties of the structures, which can be derived from microCT images.

Despite their potential, microFE models have three key limitations: (1) their displacement and strain field predictions must be validated against experimental data; (2) boundary conditions must be defined to simulate realistic loading conditions; and (3) these large‐scale microFE models are primarily used within the linear regime (before permanent deformations occur during failure) and nonlinear models are too computationally expensive for modelling large portions of tissues or organs. To address the first two limitations, a coupling between microFE and DVC models has been proposed for various bone applications. In these approaches, time‐lapsed mechanical tests are conducted in situ within a microCT system. The undeformed image is converted into a microFE model and DVC data are then used to interpolate realistic boundary conditions for the model. The microFE and DVC results in the middle portion of the specimen are compared to assess the ability of the microFE model to predict local displacements (i.e., the primary endpoint, as it is less influenced by uncertainties compared to strains) at the 40–50 voxel spatial resolution of DVC. This allows the microFE model to estimate local strain at the voxel level.

It should be noted that, currently, there is no experimental method to accurately evaluate deformation at the voxel level. Therefore, the predictions made by microFE models at this scale may be affected by increased predictive error due to the propagation of errors from larger scales. Nevertheless, this represents the best available approach at this stage. With this coupling method, linear microFE models driven by DVC‐derived boundary conditions have been shown to be accurate in predicting local displacements at the 40–50 voxel scale for various types of mineralised structures, including trabecular bone,^7^, ^63^, ^64^ bone‐cement interface,^65^ vertebral bodies with^13^ and without^12^ lesions, and mouse tibia.^66^ In order to tackle the third limitation, nonlinear microFE models have been recently shown to improve the predictions of local displacement in trabecular bone specimens tested beyond yield.^51^, ^67^ Similarly, linear and nonlinear FE models based on lower resolution CT images (resembling clinical CT images) have been challenged against DVC measurements, showcasing the limitations and potential of this computational modelling approach.^68^

Moreover, the combination of DVC and microFE models has been used to better understand the mechanical properties of tissues of interest. For example, performing mechanical tests on individual trabeculae to determine their elastic moduli is extremely challenging. The DVC‐microFE coupling has been employed to overcome this difficulty,^69^ even though the usage of in situ mechanical testing using SR‐microCT may have biased the range of found elastic moduli due to induced radiation damage.^1^ Another example of DVC and FE model coupling (specifically homogenised FE models) has been used to investigate the bone density–modulus relationships for the human vertebral body, leveraging DVC data to evaluate accurate boundary conditions for the FE models.^70^

Despite the progress in coupling DVC and FE models, several challenges remain: (i) DVC assessment at the voxel level is still not sufficiently accurate, and further development is needed. This may include integrating the mechanical properties of the tested structure by incorporating FE solvers directly within the DVC framework as previously presented for metamaterials.^71^ (ii) The validation of microFE models at the voxel level remains impossible. However, a potential solution for future applications could involve multiscale DVC approaches, using higher‐resolution images for DVC and lower‐resolution images for microFE. (iii) More studies are required to extend the assessment of local deformation in larger specimens, particularly using nonlinear microFE models coupled with DVC. (iv) Constitutive laws for complex biological tissues could be further refined by combining DVC and FE modelling techniques.

### 4.3 DVC in design of tissue engineering materials and implant–tissue stability

DVC is a unique technique for assessing the biomechanical properties of biomaterials designed for tissue engineering. In hard tissue engineering, the goal is to create scaffolds that mimic the structure and mechanical properties of natural bone. Magnesium (Mg) alloys are promising materials for bone regeneration due to their biodegradability and osteoconductive properties. These alloys must possess sufficient mechanical strength and morphological similarity to native bone to provide adequate support and facilitate bone bridging. To evaluate the suitability of Mg‐based scaffolds, a combination of techniques was employed, including in situ CT mechanics, DVC, electron microscopy, and nanoindentation.^21^ Combining CT with in situ mechanical testing and DVC helps to understand bone‐biomaterial interactions and the local mechanics of bone regeneration during healing.^72^, ^73^ This provides valuable insights for developing and optimising novel osteoregenerative biomaterials. However, the high degradation rate of magnesium alloys can compromise their mechanical integrity. To address this issue, analysing the degradation pattern of coated Mg‐based scaffolds immersed in simulated body fluid, together with DVC, is particularly useful for evaluating the local mechanics and morphological changes in such scaffolds.^74^

In soft tissue engineering, the focus is on creating scaffolds that mimic the complex hierarchical structure and mechanical properties of tissues such as tendons and ligaments (T/L). Electrospinning is a versatile technique for producing nanofibrous scaffolds with controlled architecture and composition.^75^ In this sense, electrospun scaffolds can be designed to promote cell proliferation and extracellular matrix (ECM) production, essential for tissue regeneration.^76^ Mechanical strains play a crucial role in guiding cell behaviour and ECM deposition and DVC provides a three‐dimensional map of the strain distribution within the scaffold. This is particularly important for hierarchically organised scaffolds, which consist of millions of nanofibres that slide against each other under load. DVC can reveal how these nanofibres interact and how their arrangement affects the overall mechanical properties of the scaffold. A recent study explored the use of electrospun hierarchical scaffolds made of poly‐L(lactic) acid/collagen blend to mimic the multiscale structure of T/L tissues, where DVC was used to measure the full‐field strain distribution of such structures.^77^ By analysing the strain distribution, it is also possible to optimise the scaffold's architecture to promote uniform cell seeding, migration, and differentiation. Furthermore, DVC can be combined with advanced imaging techniques, such as SR‐microCT, to achieve higher resolution and capture finer details of the scaffold's microstructure.^78^ This approach enables the visualisation and measurements at the nanofibre level, providing a better understanding of the scaffold's mechanical behaviour.

Another application of DVC is to study orthopaedic implant–tissue positioning and primary stability. Thanks to the unique ability of DVC to evaluate internal displacements and deformations of the tested specimens, it is an attractive technique to understand the micromotions between the prosthesis and the tissue where it has been implanted. In a recent study, DVC has been applied to three human cadaver femurs implanted with the femoral component of a cementless total hip replacement to evaluate the effect of implanting on the compaction of the bone around the implant.^79^ This pilot study highlighted the heterogeneous bone compaction around the implant due to the local bone density and plastic deformation of the bone, and the contact area between the implant and the bone. With a similar approach, Wearne et al. (2024) studied the interference fit of cementless proximal tibial implants in seven cadaver tibiae, highlighting the heterogeneous bone‐implant interference across the pegs and keel of the studied prosthesis and the needs of this complex tests to better understand implant positioning.^80^ The same group has also evaluated with DVC the local deformation and the residual strains of the trabecular bone around the above‐mentioned tibia implant under different levels of compressive loads.^81^ They found that the measured large strain values and residual strain localised mainly within 1.56 mm from the bone‐implant boundary, highlighting the importance of good bone quality in that area for a good implant stability. In another study, DVC has been used to evaluate the mechanical behaviour of cemented glenoid implants under a typical load experience in vivo during glenoid lift‐off, with particular focus on the deformation around the implant peripheral peg in osteoarthritic tissue.^82^ They found that regions closer to the glenoid implant backside are subjected to higher strains and are therefore more likely to initiate failure. Similar to previous analyses, they also found that the high strains localised in the trabecular bone directly in contact with the bone cement. These studies highlight how DVC can be used in orthopaedic biomechanics to better characterise the deformation of the bone tissues in shoulder, hip and knee arthroplasty.

While DVC is a powerful tool, it is not without limitations also in this field. The resolution of microCT systems may not always be sufficient to resolve individual nanofibres or the internal micro‐/nanoporosities within the scaffold. Additionally, the step‐wise nature of in situ tests can lead to partial relaxation of the scaffold due to the viscoelasticity of polymeric materials. Moreover, the artefacts introduced by metallic implants could affect the DVC outputs and, considering that the highest strains localise around the implant, an increase in the spatial resolution of the DVC would improve our understanding of what happens at the interfaces between implant, cement and bone.

Despite these challenges, DVC is transforming the field of biomaterials and orthopaedic implant design by providing unprecedented insights into the mechanical behaviour of scaffolds for hard and soft tissue regeneration and in bone‐implant interfaces. By combining DVC with advanced imaging and computational modelling techniques, it is possible to create more effective and biomimetic scaffolds and better orthopaedic implants that promote tissue regeneration and improve patient outcomes.

### 4.4 More efficient DVC analysis supported by data‐driven approaches

Imaging‐based measurements showed great potential to improve the understanding of structure‐function relationship in musculoskeletal tissues. DVC from microCT applied to bone, tooth and other biological structures have massively evolved over the past 20 years of research and are now consolidated methods, used by many researchers in the field of biomechanics. However, being experimental in nature, they don't possess any predictive ability. In this sense, and other than more traditional coupling with finite element simulation, the possibility of integration with AI‐based models is very attractive.

The emergence of AI‐based models, have unlocked a new era for agile resolution of intricate tasks and already demonstrated prowess in classifying stages of bone tissue deformation leading to fractures, employing high‐resolution tomography and in situ mechanics.^83^ Recent advances have seen physics‐informed neural networks leveraged to predict full‐field data for many processes, including crack propagation and mechanical fields within a structure.^84^ In this regard, deep learning models equipped with the capacity to predict physical fields, such as stress or strain, directly from images encapsulating geometry and microstructure information have emerged, also using partial data. To such extent, ongoing investigations are focussing on the development of a new approach for data‐driven image mechanics (D^2^IM) to predict displacement and strain fields directly from the greyscale content and texture of undeformed CT images, through a synergistic integration of DVC‐measured full‐field displacements and deep learning.^20^ Consequently, the potential of these methods to exploit the rich greyscale content and measured full fields from CT images of complex biological structures, such as hard tissues, will be one of the main topics in years to come.

## 5 OPEN CHALLENGES AND OPPORTUNITIES FOR FUTURE RESEARCH

DVC has revolutionised the ability to map internal deformations of musculoskeletal tissues, yet several key challenges remain to unlock its full potential (see outstanding questions box below). Large, geometrically complex specimens, such as joints or spinal segments, demand a multiscale DVC framework capable of capturing both global motion and localised tissue strains. The intrinsic complexity of multitissue structures (e.g., bone–cartilage, vertebra–IVD) calls for advanced multimodal imaging protocols, overcoming current limitations in tissue staining, irradiation‐induced deterioration and visualisation. In vivo applications present unique potential but also constraints, including reduced imaging resolution and the necessity to prioritise displacement tracking over fine strain measurements. Future developments must address low‐signal environments and dynamic, weight‐bearing conditions. Extending DVC to capture viscoelastic responses at physiologically relevant rates and durations will be crucial for modelling real‐world tissue behaviour. A new generation of physics‐enhanced DVC algorithms, integrating material properties directly into correlation processes, will enable more accurate interpretation of complex biomechanical phenomena. Benchmarking remains a major barrier: standardised open‐access datasets and rigorous cross‐validation of algorithms are constantly needed to drive reproducibility and innovation. Looking beyond current horizons, the integration of DVC with machine learning and data‐driven approaches offers a transformative leap. AI‐powered models can enhance feature detection, automate correlation across scales, and predict internal deformation fields in near real time. Ultimately, these developments pave the way towards novel DVC applications, enabling personalised biomechanical assessment of tissues. This vision holds the promise of tailoring treatments based on patient‐specific data, unlocking a new era of precision medicine in orthopaedics, rehabilitation, and tissue engineering.


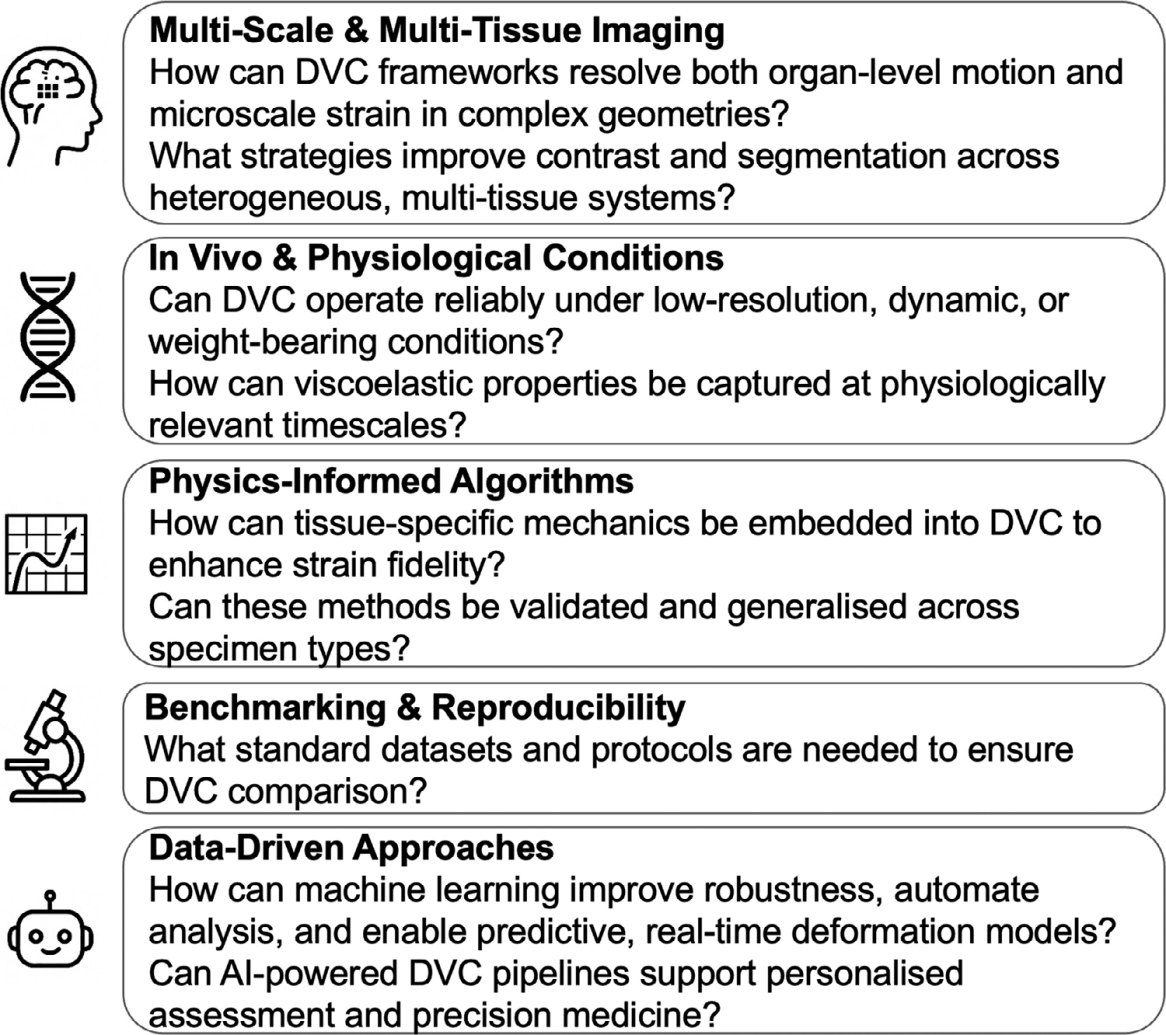


## Data Availability

Data sharing not applicable to this article as no datasets were generated or analysed during the current study.
